# Nonlinear association between body fat percent and type 2 diabetes risk in Japanese adults: a retrospective cohort study

**DOI:** 10.3389/fendo.2026.1916092

**Published:** 2026-08-11

**Authors:** Jiaqin He, Wei Sha

**Affiliations:** 1 Department of Endocrinology, Xuyi Clinical College, Medical College of Yangzhou University, Huai’an, Jiangsu, China; 2 Department of Urology, Xuyi Clinical College, Medical College of Yangzhou University, Huai’an, Jiangsu, China

**Keywords:** body fat percentage, cohort study, insulin resistance, nonlinearly, type 2 diabetes

## Abstract

**Background:**

Type 2 diabetes (T2D) has emerged as a major global public health challenge, with dyslipidemia playing a pivotal role in its pathogenesis. Body fat percentage (BFP), a direct measure of adiposity, surpasses body mass index (BMI) in identifying metabolic risk; however, its prospective association with incident T2D remains unclear.

**Objective:**

To systematically investigate the association between BFP and the risk of incident T2D and to identify potential nonlinear thresholds.

**Methods:**

A large-scale retrospective cohort study was conducted involving 15,464 Japanese adults with a mean follow-up of 6.05 years. Cox proportional hazards regression models were employed to estimate hazard ratios (HRs), and restricted cubic spline analyses were performed to assess nonlinear relationships.

**Results:**

BFP was identified as an independent risk factor for T2D. After comprehensive adjustment for confounders, each 1% increase in BFP corresponded to a 6% increase in T2D risk (HR = 1.06, 95% CI: 1.04–1.09). Crucially, restricted cubic spline analysis revealed a significant nonlinear "hockey-stick" pattern, identifying an inflection point at 21.65%. Below this threshold, the risk rose sharply with increasing BFP (HR = 1.18, 95% CI: 1.03–1.36); above it, the upward trend plateaued. This association remained robust in subgroups with normal waist circumference and absence of fatty liver disease, underscoring BFP's unique utility in detecting normal-weight obesity.

**Conclusion:**

BFP serves as a crucial predictor of T2D. Maintaining BFP below 21.65% may represent a novel target for personalized diabetes prevention strategies.

## Background

Diabetes has become one of the most challenging public health issues worldwide, with its continuously rising prevalence imposing a significant disease burden and economic costs (1). Obesity is widely recognized as the primary modifiable risk factor for type 2 diabetes, and elucidating the precise relationship between the two is crucial for early disease prevention and risk stratification (2). In clinical practice and epidemiological research, body mass index (BMI) is often used as a simplified indicator for assessing obesity (3, 4). However, it fails to accurately reflect the true distribution and level of body fat content in individuals (5). Body fat percent (BFP), as a direct measure quantifying the proportion of body fat, demonstrates superior efficacy compared to BMI in identifying metabolic risks (6, 7). It is particularly valuable in revealing the high-risk population of normal-weight obesity.

Numerous cross-sectional studies have confirmed a significant positive correlation between BFP and diabetic status (8, 9). However, evidence from prospective cohort studies, particularly regarding the specific form of this relationship, remains insufficient. Most existing research is based on the assumption of a linear relationship, which may obscure underlying complex biological mechanisms. Physiologically, excessive adipose tissue accumulation drives a state of dyslipidemia characterized by elevated circulating free fatty acids (FFAs) and altered adipokine profiles. This milieu induces lipotoxicity, where ectopic lipid deposition and chronic low-grade inflammation—mediated via the activation of Toll-like receptor 4 (TLR4) and nuclear factor-kappa B (NF-κB) signaling pathways, as well as endoplasmic reticulum stress—collectively impair pancreatic β-cell function and promote systemic insulin resistance (10, 11). Conversely, excessively low body fat may lead to insufficient metabolic reserves. Both scenarios support the hypothesis of a potentially nonlinear (e.g., U-shaped or J-shaped) association between BFP and diabetes risk. Clarifying the form of this relationship, especially identifying potential risk inflection points, is crucial for establishing precise body fat management targets.

Therefore, this study aims to thoroughly investigate the prospective association between baseline BFP and new-onset diabetes through a large-scale retrospective cohort. We not only utilized a multivariable Cox regression model to assess the effect of BFP as an independent risk factor for diabetes but also focused on employing advanced statistical methods, such as restricted cubic splines, to rigorously examine and characterize the nonlinear relationship between them. This study successfully confirms that BFP is an independent risk factor for diabetes onset and, for the first time in a large-scale population, reveals a significant nonlinear association with a clearly defined risk inflection point. Further subgroup analyses validate the stability of this association across different population subgroups. These findings provide new high-level evidence for understanding the complex relationship between body fat and diabetes risk and will offer critical scientific basis for developing personalized diabetes prevention strategies in the future.

## Methods

### Data sources and study cohorts

Dryad (www.datadryad.org) is an international open-access data repository designed to provide researchers worldwide with a reliable platform for storing, sharing, and long-term preservation of research data (12). In accordance with Dryad’s terms of service, users are permitted to conduct secondary analyses of data available on the platform, provided that such analyses do not infringe upon the intellectual property rights of the original authors. For the purposes of this research, we utilized the raw data contributed and made accessible by Professor Okamura’s research team (13).

The NAGALA cohort was established by Professor Okamura in 1994 at Murakami Memorial Hospital in Japan, primarily enrolling members of the general population who underwent health checkups at the hospital’s health screening center. All participants were required to undergo 1 to 2 health checkups annually at Murakami Memorial Hospital. Through the systematic collection and analysis of participants’ health checkup data, this study prospectively investigated risk factors influencing the onset and progression of various chronic diseases, including diabetes and non-alcoholic fatty liver disease.

**Figure 1:**
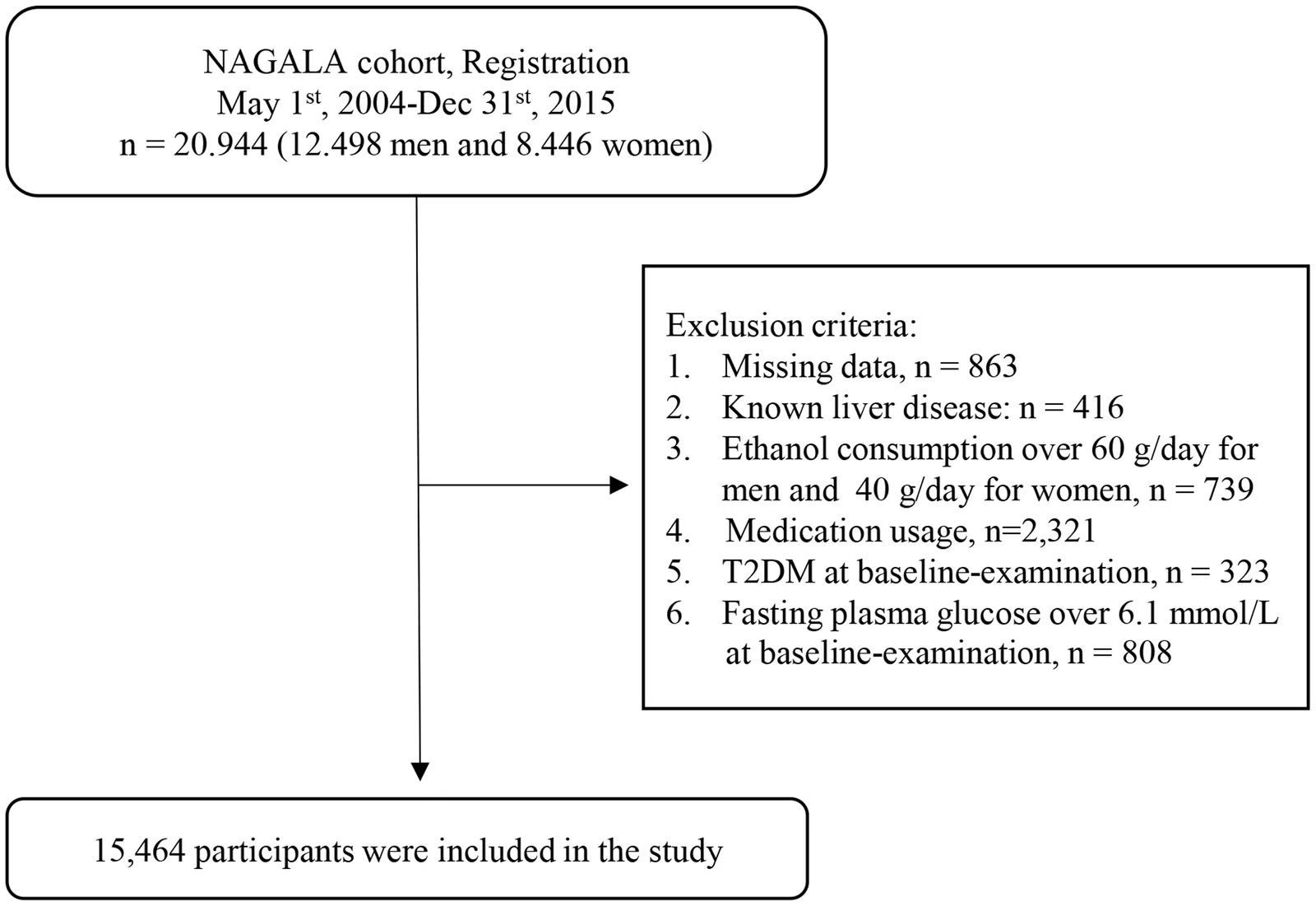
Flowchart for screening research participants.

The baseline cohort included 20,944 Japanese adults who underwent at least two health checkups between 2004 and 2015. Exclusion criteria were as follows: (1) presence of alcoholic fatty liver disease; (2) evidence of viral hepatitis at enrollment (positive hepatitis B surface antigen or positive hepatitis C antibody); (3) current use of relevant medications at baseline; (4) having diabetes or fasting plasma glucose (FPG) ≥6.1 mmol/L; (5) having a habit of regular alcohol consumption; (6) missing data on covariates. After applying the above exclusions, a total of 15,464 participants were included in the analysis. The complete screening process is shown in **Figure 1**.

Informed consent was obtained from all participants for the original study, authorizing the use of their data for scientific research. Furthermore, the study was approved by the Ethics Committee of Murakami Memorial Hospital. As a *post-hoc* analysis of the NAGALA longitudinal cohort, this study aimed to investigate the association between body fat percentage and diabetes. The study protocol was approved by the Ethics Committee of the Yangzhou university medical college xuyi clinical college. The study was conducted in accordance with the ethical principles outlined in the Declaration of Helsinki and complied with the guidelines of the Strengthening of Standards for Reporting Observational Studies in Epidemiology (STROBE).

### Data acquisition

The NAGALA database contains detailed baseline data for all participants, including basic demographic information (gender, age), medical history, and medication use. Standardized questionnaires were used to assess participants’ lifestyle characteristics, including smoking and drinking habits, as well as exercise habits. Participants were classified into three categories based on smoking habits: never smokers, former smokers, and current smokers. Alcohol consumption was categorized into four groups based on alcohol intake during the previous month. Physical activity was defined as engaging in physical exercise at least once a week. Participants’ height, weight, waist circumference (WC), and blood pressure were measured using standardized methods in a quiet environment. After fasting for at least 10 hours, venous blood was collected from each participant by a professional nurse and subsequently analyzed using an automated biochemical analyzer to measure total cholesterol (TC), triglycerides (TG), HDL-C, fasting plasma glucose (FPG), hemoglobin A1c (HbA1c), aspartate aminotransferase (AST), γ-glutamyltransferase (GGT), and alanine aminotransferase (ALT). Gastroenterologists diagnosed fatty liver based on the results of abdominal ultrasound examinations performed on the patients; diagnostic criteria included deep attenuation, hepatic-renal echogenic contrast, hepatic brightness, and vascular blurring (13).

### Definition

BFP = 1.2 × BMI + 0.23 × age − 10.8 × sex − 5.4, where sex = 1 for men and 0 for women (14). Values are expressed as percentages (%). It is important to note that while dual-energy X-ray absorptiometry (DXA) or bioelectrical impedance analysis (BIA) provides precise measurements of body composition, their application in large-scale epidemiological screenings is often limited by high costs and technical complexity. In this study, BFP was estimated using a validated formula specifically derived for Asian populations, which allows for efficient assessment of adiposity in large cohorts and facilitates potential implementation in resource-limited primary care settings (15).

Diabetes was defined as participants with HbA1c ≥6.5%, FPG ≥7 mmol/L, or self-reported diabetes during follow-up. Normal waist circumference was defined as < 85 cm in men and < 80 cm in women, according to the Japanese obesity diagnostic criteriaand IDF Asia–Pacific recommendations.

Hypertension was defined according to the Japanese Society of Hypertension Guidelines for the Management of Hypertension (JSH 2019/2024) as follows: Systolic blood pressure (SBP) ≥ 140 mmHg, or Diastolic blood pressure (DBP) ≥ 90 mmHg, or Self-reported current use of antihypertensive medication (16).

### Statistical analysis

This study divided participants into four groups based on BFP quartiles. Continuous variables are expressed as mean ± standard deviation (x¯ ± s). Categorical variables are presented as frequency (n, %). Continuous variables were compared using one-way analysis of variance or the Kruskal-Wallis H test. For categorical variables, differences were assessed using chi-square tests or Fisher’s exact tests. Cumulative incidence rates of diabetes were compared using the Kaplan-Meier method, and the log-rank test was employed to analyze hazard ratios (HR) for diabetes risk across different BFP groups.

To investigate the relationship between BFP and diabetes risk, we conducted multivariate Cox proportional hazards regression analyses. In this study, we employed three distinct Cox proportional hazards regression models: an unadjusted model, a partially adjusted model (adjusting for hypertension, fatty liver, waist circumference, physical activity, alcohol consumption, and smoking status), and a fully adjusted model (adjusting for hypertension, fatty liver, waist circumference, physical activity, alcohol consumption, smoking status, ALT, AST, GGT, HDL-C, TC, and TG). Additionally, multiple sensitivity analyses were conducted to validate the robustness of the results. This study did not include baseline fasting plasma glucose (FPG) and glycated hemoglobin (HbA1c) in the final model for adjustment. This decision was primarily based on etiological reasoning: existing physiological evidence suggests that excessive fat accumulation triggers insulin resistance, which in turn leads to elevated blood glucose levels and ultimately progresses to diabetes. In this pathophysiological pathway, FPG and HbA1c function as mediators between the exposure (BFP) and the outcome (type 2 diabetes), rather than as confounders. Over-adjusting for such mediators located downstream in the causal pathway within the model may dilute the total effect of BFP on the onset of diabetes, thereby underestimating its independent hazard. Therefore, we chose to adjust for upstream lifestyle and metabolic comorbidity factors in order to more accurately reflect the effect size of BFP as an exposure factor. In addition, multicollinearity was assessed for all covariates included in the model by calculating the variance inflation factor (VIF). The results showed that the VIF values for all variables were less than 5, with the highest value being 4.2, indicating that there was no severe multicollinearity among the variables and that the model was stable.

We conducted multiple sensitivity analyses to confirm the robustness of our findings. First, we excluded participants whose BFP values deviated from the mean by more than 3 standard deviations to reduce the impact of outliers. Subsequently, we performed stratified analyses based on specific metabolic health indicators. The analyses were restricted to individuals with (I) a normal waist circumference (men < 85 cm, women < 80 cm) to define “normal-weight obesity”; (II) the absence of fatty liver to determine whether BFP predicts diabetes independently of hepatic steatosis; and (III) the absence of hypertension to minimize confounding from hemodynamic and sympathetic overactivity. These specific factors were selected to disentangle the direct effects of obesity from downstream complications of the metabolic syndrome continuum. Furthermore, the potential impact of unidentified confounders on the association between BFP and diabetes risk was assessed by calculating the E-value. The E-value is a metric used to assess the impact of unmeasured confounding factors on the results of observational studies. It represents the minimum strength of association that unmeasured confounding factors must possess to fully explain the observed association between exposure and outcome.

We employed cubic spline functions and smooth curve fitting to explore potential nonlinear relationships between BFP and diabetes. A segmented Cox proportional hazards regression model was constructed using a recursive algorithm to identify inflection points and analyze data on either side of these points separately. Finally, the optimal model explaining the association between BFP and diabetes risk was determined via the log-likelihood ratio test. Subgroup analyses were performed using stratified Cox proportional hazards models for different population subgroups (age, sex, BMI, hypertension, smoking/drinking status, and fatty liver). Interaction effects among subgroups were assessed via likelihood ratio tests. P-values < 0.05 were considered statistically significant. All statistical analyses were conducted using Empower Stats (R) software (www.EmpowerStats.com, X&Y Solutions, Inc., Boston, MA) version 6.0 and R software version 3.6.1 (http://www.r-project.org, R Foundation).

## Results

### Baseline characteristics of participants

This study included 15,464 participants with a mean age of 43.71 ± 8.90 years. Females constituted 54.48% of the cohort. The BFP for all participants ranged from 9.57% to 50.03%, with a mean ± standard deviation of 25.31 ± 5.86%. All participants were divided into four groups based on BFP quartiles: (Q1 ≤ 20.93%; 20.93% < Q2 ≤ 25.14%; 25.14% < Q3 ≤ 29.23%; and Q4 > 29.23%). **Table 1** presents baseline characteristics of the study population. During a mean follow-up period of 6.05 years, 373 participants (2.41%) developed T2D. Notably, the cumulative incidence​ of diabetes increased significantly with rising BFP levels, from 1.37% in Q1 to 2.95% in Q4 (*P* < 0.001). Concurrently, Participants in the higher BFP quartiles were older and more likely to report alcohol consumption, current smoking, and physical inactivity compared with those in the lowest quartile (*P* < 0.001).

**Table 1:** The baseline characteristics of participants.

BFP quartile	Q1 (9.57-20.93)	Q2 (20.93-25.14)	Q3 (25.14-29.23)	Q4 (29.24-50.03)	P-value
Participants	3866	3866	3866	3866	
Age, year	39.58 ± 7.05	43.57 ± 9.35	43.60 ± 8.80	48.08 ± 8.12	<0.001
BMI, kg/m^2^	20.86 ± 1.82	22.38 ± 2.94	21.79 ± 3.49	23.44 ± 3.38	<0.001
WC, cm	74.89 ± 5.51	78.34 ± 9.00	74.92 ± 10.85	77.73 ± 9.62	<0.001
ALT, U/L	20.22 ± 10.36	22.49 ± 13.56	19.44 ± 19.49	17.81 ± 11.86	<0.001
AST, U/L	18.42 ± 7.14	18.99 ± 7.26	18.27 ± 11.76	17.94 ± 7.50	<0.001
Weight, kg	61.51 ± 7.09	63.71 ± 12.07	58.23 ± 13.75	59.10 ± 11.69	<0.001
GGT, U/L	21.50 ± 17.90	24.55 ± 22.44	18.90 ± 16.90	16.29 ± 12.99	<0.001
HDL-C, mmol/L	1.40 ± 0.36	1.37 ± 0.40	1.54 ± 0.44	1.54 ± 0.39	<0.001
TC, mmol/L	4.96 ± 0.82	5.11 ± 0.86	5.08 ± 0.85	5.36 ± 0.88	<0.001
TG, mmol/L	0.90 ± 0.60	1.05 ± 0.74	0.86 ± 0.70	0.85 ± 0.54	<0.001
HbA1c, %	5.10 ± 0.30	5.16 ± 0.32	5.17 ± 0.32	5.26 ± 0.33	<0.001
FBG, mmol/L	5.21 ± 0.37	5.23 ± 0.42	5.09 ± 0.44	5.11 ± 0.41	<0.001
HBP, mmHg	113.82 ± 12.25	116.18 ± 15.13	112.21 ± 16.16	115.78 ± 15.71	<0.001
LBP, mmHg	71.02 ± 8.74	73.33 ± 10.69	70.10 ± 11.42	71.87 ± 10.71	<0.001
BFP, %	18.05 ± 2.13	23.10 ± 1.21	27.11 ± 1.16	32.97 ± 3.23	<0.001
Gender					<0.001
Female	42 (1.09%)	866 (22.40%)	2552 (66.01%)	3574 (92.45%)	
Male	3824 (98.91%)	3000 (77.60%)	1314 (33.99%)	292 (7.55%)	
Fatty liver					<0.001
No	3486 (90.17%)	2917 (75.45%)	3148 (81.43%)	3172 (82.05%)	
Yes	380 (9.83%)	949 (24.55%)	718 (18.57%)	694 (17.95%)	
physical activity					0.595
No	3168 (81.95%)	3183 (82.33%)	3213 (83.11%)	3191 (82.54%)	
Yes	698 (18.05%)	683 (17.67%)	653 (16.89%)	675 (17.46%)	
Drinking status					<0.001
Non	2576 (66.63%)	2632 (68.08%)	3113 (80.52%)	3484 (90.12%)	
Light	609 (15.75%)	549 (14.20%)	358 (9.26%)	242 (6.26%)	
Moderate	469 (12.13%)	469 (12.13%)	295 (7.63%)	127 (3.29%)	
Heavy	212 (5.48%)	216 (5.59%)	100 (2.59%)	13 (0.34%)	
Smoking status					<0.001
Never	1461 (37.79%)	1744 (45.11%)	2601 (67.28%)	3225 (83.42%)	
Past	961 (24.86%)	1028 (26.59%)	645 (16.68%)	318 (8.23%)	
Current	1444 (37.35%)	1094 (28.30%)	620 (16.04%)	323 (8.35%)	
Diabetes					<0.001
No	3813 (98.63%)	3766 (97.41%)	3760 (97.26%)	3752 (97.05%)	
Yes	53 (1.37%)	100 (2.59%)	106 (2.74%)	114 (2.95%)	

Apparent discrepancies between BMI and BFP quartiles are attributable to the significant difference in gender distribution across quartiles.

### Incidence of diabetes

**Figure 2:**
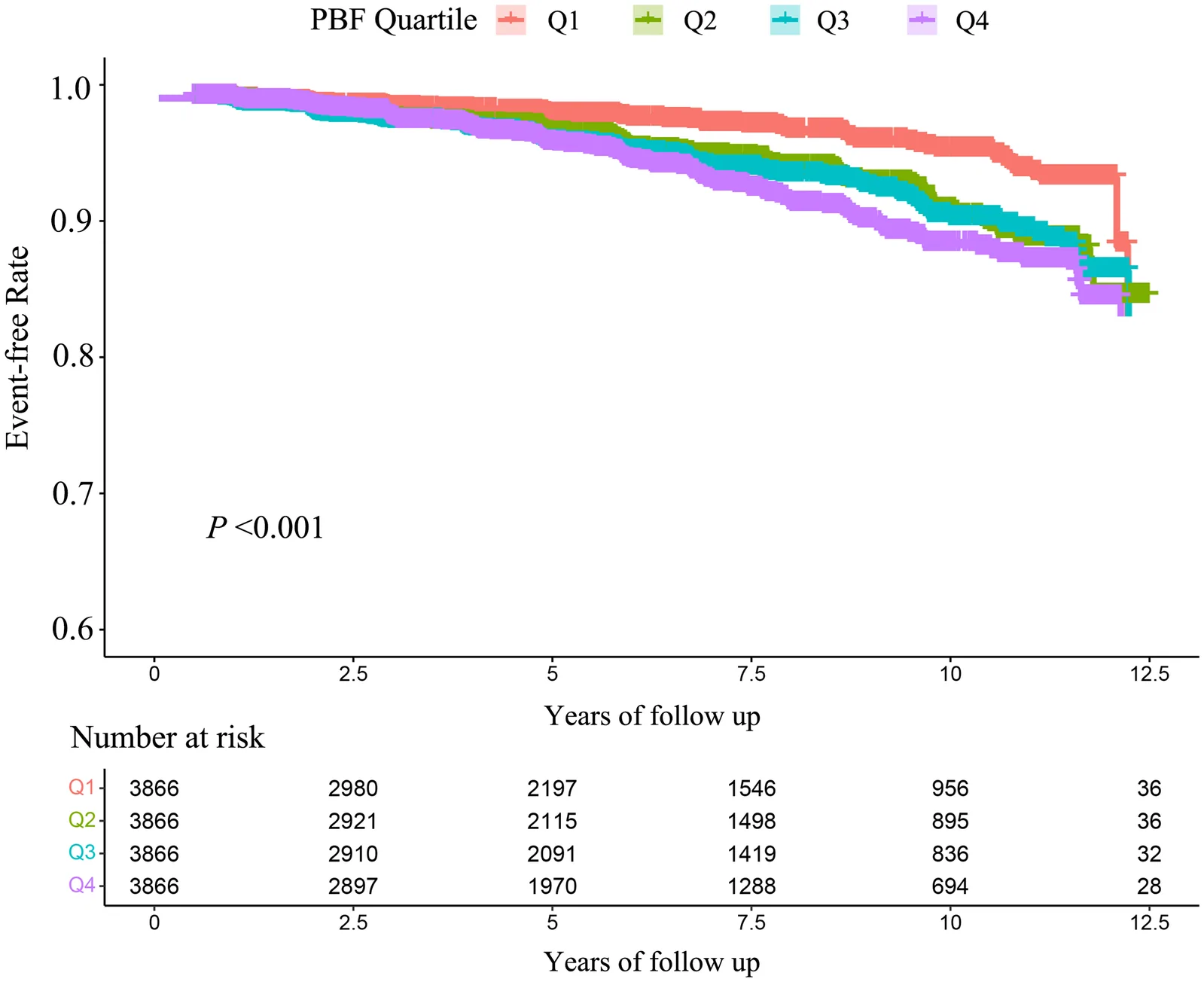
Kaplan–Meier event-free survival curve. Kaplan–Meier analysis of incident diabetes based on BFP (log-rank, *P* < 0.0001).

During follow-up, the overall diabetes prevalence among 15,464 participants was 2.41%. Participants were divided into four groups based on BFP quartiles, with prevalence rates of Q1: 1.37%; Q2: 2.59%; Q3: 2.74%; and Q4: 2.95%, *P* < 0.001 (**Figure 2**). Furthermore, the cumulative incidence rate across all subjects was 398.68 per 100,000 person-year. Cumulative incidence rates within different BFP subgroups were Q1: 226.59 per 100,000 person-year, Q2: 427.54 per 100,000 person-year, Q3: 453.19 per 100,000 person-year, and Q4: 487.41 per 100,000 person-year. Statistical analysis revealed that both the prevalence and cumulative incidence of diabetes were significantly higher in the high BFP group compared to the low BFP group (*P* < 0.001) (**Table 2**).

**Table 2:** Incidence rate of type 2 diabetes.

BFP (%)	Participants (n)	Prediabetes events (n)	Cumulative incidence (%)	Per 100,000 person-year
Total	15464	373	2.41	398.68
Q1	3866	53	1.37	226.59
Q2	3866	100	2.59	427.54
Q3	3866	106	2.74	453.19
Q4	3866	114	2.95	487.41
P for trend		<0.001	<0.001	<0.001

### The relationship between BFP and diabetes

In this study, we employed three distinct Cox proportional hazards regression models to assess the association between BFP and diabetes (**Table 3**). First, in the unadjusted model, each 1% increase in BFP was associated with a 27% higher risk of diabetes, with a hazard ratio (HR) of 1.27 (95% confidence interval: 1.15–1.39; *P* < 0.001). In the minimally adjusted model (adjusting for waist hypertension, fatty liver, waist circumference, physical activity, alcohol consumption, and smoking status), the HR (95% CI) was 1.05 (1.03–1.07). After further adjustment for AST, ALT, GGT, HDL-C, TC, and TG, the association between BFP and diabetes remained statistically significant (HR = 1.06, 95% CI: 1.04–1.09; *P* < 0.001). Furthermore, after converting BFP into quartiles, the analysis revealed a persistent significant association between BFP and diabetes. In the fully adjusted model, the risk of diabetes in the highest BFP quartile was 108% higher than in the lowest quartile (HR = 2.08, 95% CI: 1.42–3.06; *P* < 0.001). Trend analysis demonstrated a significant increasing trend in diabetes risk with rising BFP levels (*P* < 0.001, **Table 3**).

**Table 3:** Hazard ratios for incident type 2 diabetes according to BFP in Cox proportional hazards models with different adjustments.

Exposure	Non-adjusted (HR, 95%CI, *P*)	Mini-adjusted mode (HR, 95%CI, *P*)	Fully-adjusted mode (HR, 95%CI, *P*)
BFP (%)	1.27 (1.15, 1.39) <0.001	1.05 (1.03, 1.07) <0.001	1.06 (1.04, 1.09) <0.001
BFP quartile (%)			
Q1	Reference	Reference	Reference
Q2	1.95 (1.40, 2.73) <0.001	1.08 (0.73, 1.52) 0.674	1.06 (0.75, 1.49) 0.759
Q3	2.13 (1.53, 2.99) <0.001	1.50 (1.05, 2.13) 0.025	1.48 (1.04, 2.11) 0.031
Q4	2.48 (1.79, 3.44) <0.001	1.84 (1.26, 2.69) <0.001	2.08 (1.42, 3.06) <0.001
P for trend	<0.001	<0.001	<0.001

Non-adjusted model: no adjust for other covariates; Mini-adjusted mode: adjusted for hypertension, fatty liver, waist circumference, physical activity, alcohol consumption, and smoking status. Fully-adjusted mode: adjusted for hypertension, fatty liver, waist circumference, physical activity, alcohol consumption, smoking status, ALT, AST, GGT, HDL-C, TC, and TG. HR, hazard ratios; CI, confidence interval.

### Sensitivity analysis

We conducted multidimensional sensitivity analyses to verify the stability of the association between BFP and diabetes. First, to exclude the influence of outliers, we excluded participants whose BFP values fell outside the range of ±3 standard deviations from the mean, ultimately including 15,411 participants in the subsequent analysis. After comprehensive adjustment for confounding factors, the association between BFP and diabetes risk remained statistically significant (HR = 1.07, 95% CI: 1.02–1.12; P = 0.008) (**Table 4**, Model 1). Second, among individuals with normal waist circumference (<85 cm in men and <80 cm in women). After adjusting for relevant covariates, a significant positive association persisted between BFP and diabetes risk, with an HR of 1.07 (95% CI: 1.05–1.09; *P* < 0.001) (**Table 4**, Model 2). Next, in individuals without fatty liver disease, the analysis showed that BFP remained positively associated with diabetes risk. Each 1% increase in BFP was associated with an 86% increase in diabetes risk (**Table 4**, Model 3). Furthermore, among individuals without hypertension, participants with high BFP exhibited a significantly elevated diabetes risk (HR: 2.56, 95% CI: 1.51–4.32; *P* < 0.001) (**Table 4**, Model 4). Finally, the calculated E-value of 2.86 indicated high statistical significance, suggesting that strong unmeasured confounding would be required to explain away the observed association.

**Table 4:** Relationship between BFP and diabetes in different sensitivity analyses.

Exposure	Model 1 (HR, 95%CI, *P*)	Model 2 (HR, 95%CI, *P*)	Model 3 (HR, 95%CI, *P*)	Model 4 (HR, 95%CI, *P*)
BFP (%)	1.07 (1.02, 1.12) 0.008	1.07 (1.05, 1.09) <0.001	1.05 (1.02, 1.09) 0.003	1.09 (1.05, 1.12) <0.001
BFP quartile (%)				
Q1	Reference	Reference	Reference	Reference
Q2	0.95 (0.64, 1.41) 0.792	1.70 (0.96, 3.02) 0.067	1.22 (0.77, 1.95) 0.396	1.25 (0.79, 1.96) 0.335
Q3	1.23 (1.08, 1.41) 0.002	3.34 (1.88, 5.93) <0.001	1.65 (1.18, 2.30) 0.003	1.69 (1.04, 2.76) 0.034
Q4	2.19 (1.69, 2.86) <0.001	3.81 (2.14, 6.78) <0.001	1.86 (1.07, 3.24) 0.029	2.56 (1.51, 4.32) <0.001
P for trend	<0.001	<0.001	<0.001	<0.001

Model 1 presents a sensitivity analysis of the participants after excluding BFP outliers. We adjusted hypertension, fatty liver, waist circumference, physical activity, alcohol consumption, smoking status, ALT, AST, GGT, HDL-C, TC, and TG. Model 2 was sensitivity analysis in participants with normal waist circumference. We adjusted hypertension, fatty liver, physical activity, alcohol consumption, smoking status, ALT, AST, GGT, HDL-C, TC, TG; Model 3 was sensitivity analysis in participants without Fatty liver. We adjusted hypertension, waist circumference, physical activity, alcohol consumption, smoking status, ALT, AST, GGT, HDL-C, TC, and TG. Model 4 was sensitivity analysis in participants without hypertension. We adjusted fatty liver, waist circumference, physical activity, alcohol consumption, smoking status, ALT, AST, GGT, HDL-C, TC, and TG. HR, hazard ratios; CI, confidence.

### Nonlinear association between BFP and diabetes

**Figure 3:**
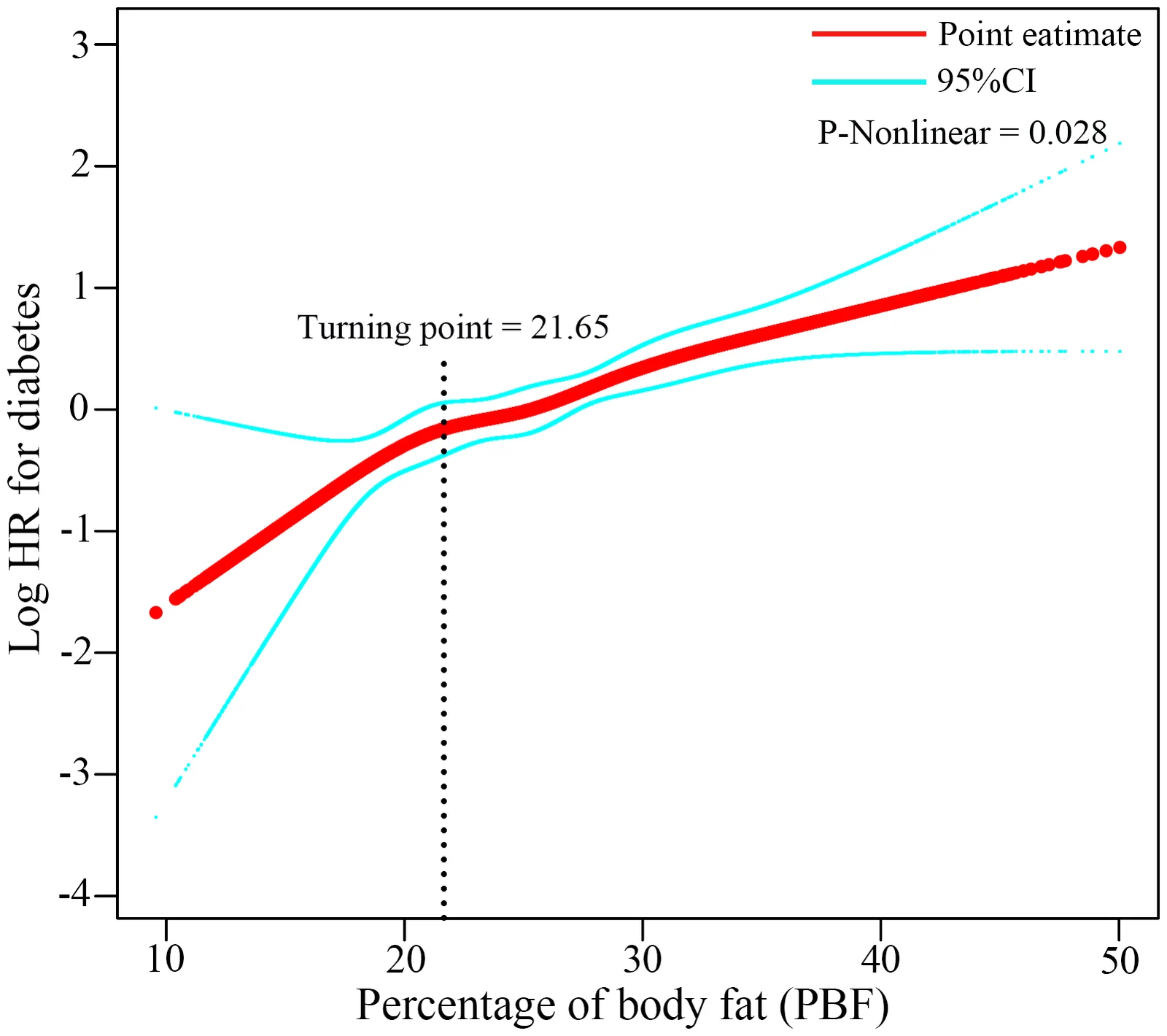
The nonlinear relationship between BFP and incident diabetes. Adjusted for hypertension, fatty liver, waist circumference, exercise habits, alcohol consumption, smoking status, ALT, AST, GGT, HDL-C, TC, and TG. HR, hazard ratios; CI, confidence interval.

The nonlinear relationship between BFP and diabetes was investigated using restricted cubic spline functions and smooth curve fitting (**Table 5**). Results from the two-stage Cox proportional hazards regression model revealed a nonlinear association between BFP and diabetes, with an inflection point at BFP = 21.65% (log-likelihood ratio test *P* < 0.001). When BFP ≤ 21.65%, BFP was positively associated with diabetes risk (HR: 1.18, 95% CI: 1.03–1.36; *P* < 0.001). Conversely, when BFP > 21.65%, the trend of increased diabetes risk slowed, with an HR of 1.04 (95% CI: 1.02–1.06; *P* < 0.001) (**Figure 3**).

**Table 5:** The result of the two-piecewise Cox proportional hazards regression model.

Incident type 2 diabetes	HR (95%CI)	P-value
Fitting model by standard Cox proportional hazards regression	1.03 (1.01, 1.04)	0.003
Fitting model by two-piecewise Cox proportional hazards regression		
Inflection points of BFP (%)	21.65	
≤ 21.65	1.18 (1.03, 1.36)	0.017
> 21.65	1.04 (1.02, 1.06)	0.002
P for log likelihood ratio test		0.028

Adjusted foe hypertension, fatty liver, waist circumference, physical activity, alcohol consumption, smoking status, ALT, AST, GGT, HDL-C, TC, and TG. Abbreviations: HR, hazard ratios; CI, confidence.

Further stratified analysis revealed that the aforementioned inflection point exhibited significant heterogeneity by gender and age.​ In the gender-stratified analysis, the risk inflection point for men was 16.08%, while for women it was as high as 33.16%, suggesting that women, due to estrogen-mediated metabolic protection, possess a stronger physiological fat buffer capacity and can maintain glycemic homeostasis even at higher body fat levels (**Table 6**). Therefore, the inflection point of 21.65% for the entire population is actually closer to the risk threshold for men. In the age-stratified analysis, the population was divided into a younger group (≤43.7 years) and an older group (>43.7 years) based on the median age (43.7 years). The results showed that the inflection point was 21.36% in the young group, whereas it shifted upward to 25.74% in the older group, reflecting a rightward shift in the threshold due to age-related declines in metabolic flexibility and the accumulation of visceral fat. Notably, the segmented Cox regression model performed significantly better than the standard linear model in the younger group (log-likelihood ratio test, *P* = 0.025), whereas this advantage was no longer significant in the older group (*P* = 0.283) (**Table 7**). These results suggest that the “threshold effect” of BFP is primarily concentrated in middle-aged individuals; in the elderly population, the risk of diabetes onset may be driven more linearly by other aging-related factors, such as sarcopenia and chronic inflammation.

**Table 6:** Gender-specific inflection points of BFP.

Gender	Female	Male
Incident type 2 diabetes	(HR, 95%CI, p)	(HR, 95%CI, p)
Fitting model by standard Cox proportional hazards regression	1.06 (0.97, 1.16) 0.2012	1.17 (1.11, 1.24) <0.0001
Fitting model by two-piecewise Cox proportional hazards regression		
Inflection points of BFP (K, %)	33.16	16.08
≤K	1.11 (1.00, 1.23) 0.0398	3.18 (0.56, 18.05) 0.1910
>K	0.84 (0.65, 1.08) 0.1687	1.17 (1.11, 1.23) <0.0001
P for log likelihood ratio test	0.031	0.028

Adjusted foe hypertension, fatty liver, waist circumference, physical activity, alcohol consumption, smoking status, ALT, AST, GGT, HDL-C, TC, and TG. Abbreviations: HR, hazard ratios; CI, confidence.

**Table 7:** Age-specific inflection points of BFP.

Age, years	≤43.7	>43.7
Incident type 2 diabetes	(HR, 95%CI, p)	(HR, 95%CI, p)
Fitting model by standard Cox proportional hazards regression	1.09 (1.06, 1.13) <0.0001	1.04 (1.01, 1.07) 0.0142
Fitting model by two-piecewise Cox proportional hazards regression		
Inflection points of BFP (K, %)	21.36	25.74
≤K	1.01 (0.88, 1.16) 0.8639	1.00 (0.93, 1.08) 0.9837
>K	1.11 (1.07, 1.14) <0.0001	1.06 (1.01, 1.11) 0.0118
P for log likelihood ratio test	0.025	0.283

Adjusted foe hypertension, fatty liver, waist circumference, physical activity, alcohol consumption, smoking status, ALT, AST, GGT, HDL-C, TC, and TG. Abbreviations: HR, hazard ratios; CI, confidence.

### Subgroup analyses and interaction tests

**Figure 4:**
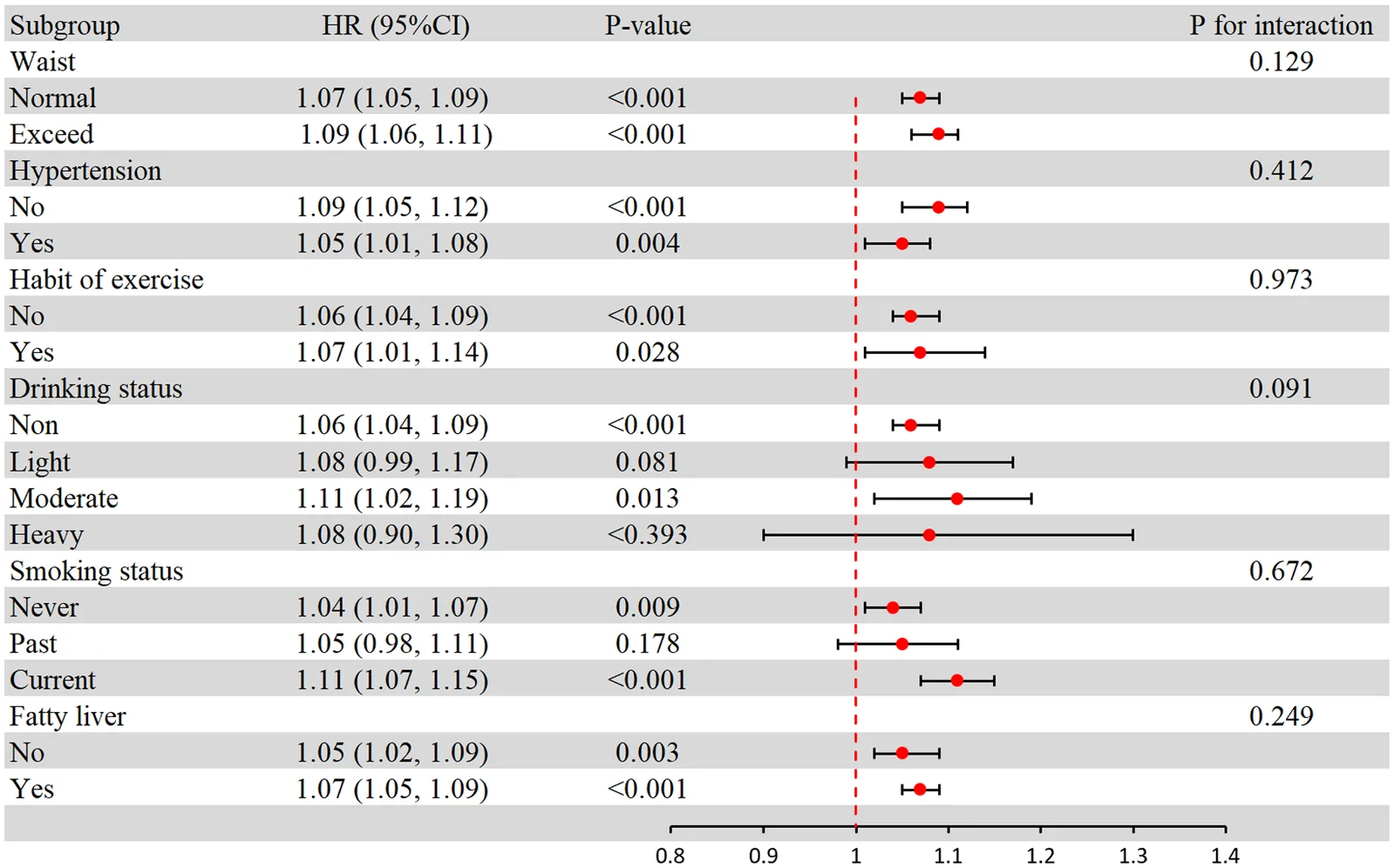
Subgroups analysis for the associations between BFP and diabetes.

We conducted subgroup analyses to assess the stability of the association between BFP and diabetes and to examine potential differences across different populations. Stratification factors included waist circumference, blood pressure, alcohol consumption status, smoking status, fatty liver, and exercise habits. Notably, the association between BFP and diabetes risk was more pronounced in subgroups with excessive waist circumference, smoking status, and fatty liver disease (all *P* < 0.05). Interaction tests indicated that the association between BFP and diabetes risk was stable and consistent across populations (all *P* > 0.05) (**Figure 4**).

## Discussion

In this large-scale retrospective cohort study based on the NAGALA cohort of Japanese adults, we found that percent body fat (BFP) is an independent risk factor for new-onset type 2 diabetes. After adjusting for traditional metabolic confounders, including blood pressure, blood lipids, liver function, and baseline blood glucose, a 1% increase in BFP was associated with a 6% increase in the risk of diabetes (HR = 1.06, 95% CI: 1.04–1.09); compared with the lowest quartile, the risk of diabetes was 108% higher in the highest BFP quartile. Notably, restricted cubic spline analysis revealed a nonlinear relationship between BFP and diabetes risk, with an inflection point at 21.65%. Furthermore, sensitivity analyses confirmed that this association remained robust in individuals with normal waist circumference, no fatty liver, and no hypertension, suggesting that BFP may provide additional predictive value beyond traditional measures of obesity.

The strength of the association between BFP and diabetes observed in this study, particularly its robustness among individuals with a normal BMI, carries significant implications for public health. For a long time, clinical and public health practices have relied excessively on BMI as the primary indicator for obesity screening and intervention, resulting in a large number of “normal-weight but high-body-fat” individuals being overlooked in primary diabetes prevention systems (17, 18). The results of this study indicate that BFP can effectively identify this hidden high-risk population. Given that Asian populations tend to have higher body fat percentages and a greater propensity for visceral fat accumulation at the same BMI levels, BFP better reflects race-specific metabolic risk characteristics compared to BMI. This finding suggests that, for the early prevention and control of diabetes, focusing solely on whether body weight is within the normal range may be insufficient; maintaining a healthy body fat ratio should become a new intervention target.

In recent years, a growing body of evidence has shown that percent body fat (BFP) not only plays a key role in impaired glucose metabolism but is also closely associated with various metabolic diseases. Cross-sectional and cohort studies have consistently found that elevated BFP is significantly associated with conditions such as hypertension, non-alcoholic fatty liver disease, dyslipidemia, and hyperuricemia (19–21); it can even identify a higher risk of metabolic syndrome in individuals with normal BMI. Particularly in studies on non-alcoholic fatty liver disease, BFP is considered a more sensitive indicator of hepatic lipid accumulation and systemic fat overload than traditional measures such as BMI and waist circumference. Although the association between BFP and various metabolic diseases has gradually been elucidated, prospective evidence regarding its role in the onset of diabetes remains relatively limited. Furthermore, most studies assume a linear relationship between body fat and disease risk, overlooking potential phasic changes and threshold effects during the accumulation of body fat (22). Unlike previous studies based on BMI or waist circumference, this study is the first to employ a restricted cubic spline model in a large-scale Japanese adult cohort to systematically investigate the dose–response relationship between BFP and new-onset diabetes, identifying a clear inflection point (21.65%). The results indicate that BFP is not only an independent risk factor for diabetes but also retains significant predictive value among individuals with normal waist circumference, no fatty liver, and no hypertension, thereby overcoming the limitations of traditional obesity indices.

From a pathophysiological perspective, body fat percentage (BFP) is not only a quantitative measure of fat content but also a key indicator of systemic energy metabolism imbalance and the functional state of adipose tissue (23). Excess body fat, particularly visceral fat—which is highly metabolically active, sensitive to catecholamines, and prone to lipolysis—can disrupt insulin signaling through various mechanisms, including lipotoxicity, chronic low-grade inflammation, and dysregulation of adipokine secretion, ultimately leading to insulin resistance and pancreatic β-cell failure (24). This study is the first to identify an inflection point of 21.65% between BFP and diabetes risk in a large-scale cohort; this finding is of great significance for understanding the phasic biological effects during the process of body fat accumulation. At the stage of lower BFP (≤21.65%), the expansion capacity of adipose tissue is often not yet fully established, and fat storage space is relatively limited; excess energy is more likely to spill over in the form of free fatty acids into insulin-sensitive organs such as the liver, skeletal muscle, and pancreas, leading to ectopic lipid deposition (25). This early lipotoxic environment can rapidly activate inflammatory pathways such as IKKβ/NF-κB and JNK, disrupting the IRS-1/PI3K/Akt insulin signaling cascade, thereby exhibiting a steep slope of risk increase within this range (26). This also explains why, even in individuals with normal BMI, once BFP exceeds this threshold, their risk of diabetes is no lower than that of typically obese individuals—a metabolic high-risk characteristic known as “normal-weight obesity.” When BFP exceeds 21.65%, the upward trend in diabetes risk slows but does not disappear; this nonlinear characteristic may result from the combined effects of multiple mechanisms. On the one hand, under long-term positive energy balance, adipose tissue may undergo adaptive remodeling through adipocyte hypertrophy and hyperplasia to some extent, thereby increasing lipid storage capacity and temporarily mitigating the acute lipotoxic shock caused by ectopic fat overflow (20). On the other hand, once insulin resistance is fully established, the marginal impairment of insulin sensitivity caused by further increases in body fat gradually weakens (27). Furthermore, the high BFP phase is often accompanied by compensatory hyperinsulinemia, allowing blood glucose levels to remain relatively stable over an extended period (28). Consequently, the immediate risk associated with continued fat accumulation manifests statistically as a flattening of the slope. Concurrently, adipose tissue under long-term high-fat conditions often exhibits macrophage infiltration, fibrosis, and hypoxia, leading to decreased adiponectin levels, leptin resistance, and sustained release of inflammatory factors (29–31). This state of slowed metabolism causes the risk curve to level off, but this does not mean that the risk of diabetes has been eliminated. It is worth noting that this study did not observe a protective association between low BFP and diabetes; therefore, it does not support the traditional U-shaped or J-shaped hypotheses but is more consistent with a “piecewise linear (hockey-stick)” model. This model emphasizes that once body fat exceeds the critical threshold of 21.65%, the body enters a new, persistently high-risk metabolic steady state. Even with further increases in body fat, the relative risk no longer increases exponentially, but the absolute risk remains consistently high. It is worth emphasizing that the core reason BFP offers predictive value beyond that of BMI and waist circumference lies in its ability to distinguish between fat mass and lean body mass, thereby identifying the specific phenotype known as sarcopenic obesity. BMI cannot distinguish whether weight gain stems from fat accumulation or muscle gain, while waist circumference, although indicative of abdominal obesity, overlooks the heterogeneity between subcutaneous and visceral fat (32, 33). In this study, BFP remained significantly associated with diabetes even among individuals with normal waist circumference, likely reflecting a pathological state of latent muscle loss accompanied by relative fat gain in this population. Muscle tissue is a key organ for glucose uptake and utilization; sarcopenia reduces basal metabolic rate and insulin sensitivity, creating a synergistic effect with the lipotoxicity of adipose tissue (34). Therefore, BFP effectively encodes dual information regarding both “diabetogenic fat load” and “loss of protective lean body mass”—a level of complexity that single-dimensional indices (such as BMI or WC) struggle to capture. In future diabetes risk assessment models, integrating BFP with muscle mass indices (such as the skeletal muscle index) may further enhance the accuracy of risk stratification.

The findings of this study have significant clinical and public health implications. A BFP of approximately 21.65% is expected to serve as a practical cutoff for diabetes risk stratification in Asian populations. For individuals with BFP below this threshold, particularly those with normal BMI but elevated BFP, even a small increase in body fat can lead to a significant rise in diabetes risk. Therefore, these individuals should be prioritized for lifestyle interventions, including controlling caloric intake and increasing aerobic and resistance exercise to maintain lean body mass and reduce fat mass (35). Subgroup analyses further revealed that the association between BFP and diabetes was more pronounced in individuals with abdominal obesity, smokers, and those with fatty liver disease, suggesting that the combined assessment of BFP with waist circumference or hepatic fat deposition could further enhance risk identification. At the same time, the independent predictive value of BFP in individuals with normal waist circumference and no fatty liver disease underscores its irreplaceable complementary role in traditional obesity assessment systems. Compared with BMI, BFP can more directly quantify fat burden, thereby providing a basis for setting individualized body composition management goals. Future interventional studies are needed to verify whether maintaining BFP below the inflection point can effectively delay or prevent the onset of diabetes.

It is worth noting that although the interaction test showed that gender did not significantly modify the overall effect of BFP on diabetes risk (interaction term *P* = 0.225), and stratified analysis confirmed that the effect was significant in both men (HR = 1.13) and women (HR = 1.10), this does not support the use of a uniform absolute cutoff point. Since the BFP calculation formula incorporates a gender parameter, and given that women in the Japanese population generally have higher physiological body fat than men, using overall quartiles based on the entire population (e.g., women account for 92.45% of the Q4 group) has limitations in terms of biological interpretation. Therefore, we further identified gender-specific risk inflection points, revealing a significant gender gap: 16.08% for men versus as high as 33.16% for women. This difference provides a biologically plausible explanation for the existence of a “physiological fat buffer” in women. Estrogen-mediated metabolic protection allows women to maintain glycemic homeostasis at higher body fat levels, thereby delaying the onset of insulin resistance; in contrast, men exhibit a “threshold switch” pattern, wherein diabetes risk rises exponentially once BFP increases moderately (>16.08%). Therefore, a universal BFP cutoff (e.g., 21.65%) is clearly suboptimal in clinical practice. To achieve precise prevention, we propose gender-specific targets: men should strictly maintain their BFP within the 16%–22% range, while women (especially premenopausal individuals) can tolerate higher levels (~30%–33%). Future clinical guidelines should incorporate these specific thresholds to avoid causing unnecessary anxiety and excessive intervention in women. In addition to gender, age is also a key factor modulating this association. Analysis stratified by the median age (43.7 years) showed that the BFP risk inflection point was 21.36% in the younger group (≤43.7 years), whereas it shifted upward to 25.74% in the older group (>43.7 years); this may be related to age-related declines in insulin sensitivity and the accumulation of visceral fat. Importantly, the segmented Cox regression model significantly outperformed the standard linear model in the younger cohort (log-likelihood ratio test, P = 0.025), suggesting that the “threshold effect” of BFP is most pronounced during middle age; whereas in the elderly group, this nonlinear characteristic was no longer significant (P = 0.283), suggesting that the risk of diabetes during the aging process may gradually evolve into a linear process driven by multiple factors such as sarcopenia and chronic inflammation, thereby diluting the independent threshold effect of BFP. Therefore, the population-wide inflection point of 21.65% should be regarded as a statistical abstraction rather than a standard for clinical practice. In clinical practice, gender and age must be considered comprehensively: for middle-aged men, maintaining BFP below 22% may represent a critical window for preventing metabolic deterioration; whereas for the elderly, caution is needed regarding the risks of malnutrition or muscle loss that may result from an excessive pursuit of low body fat, and intervention targets should be appropriately relaxed. This refined, life-stage-based stratification strategy will help achieve truly precise diabetes prevention and control.

This study also has some limitations. First, as an observational study, it cannot establish causality, although we minimized the impact of confounding biases as much as possible through multivariate adjustment and E-value analysis. Second, BFP was estimated using an empirical formula rather than measured directly via dual-energy X-ray absorptiometry (DXA) or bioelectrical impedance analysis; although this formula has been validated in multiple large-scale epidemiological studies, it cannot distinguish between subcutaneous and visceral fat. However, this estimation method also has clear advantages: it is low-cost, requires no specialized equipment, and can be used for risk stratification in community health screenings where advanced imaging is unavailable. Third, the study population consisted primarily of middle-aged and young adults in Japan (mean age 43.7 years), and the sample size in the very low BFP range (<15%) was limited (accounting for only 3.2% of the cohort). This resulted in wide confidence intervals for risk estimates in this range, limiting our ability to accurately assess the risks associated with very low body fat and weakening the generalizability of the study’s conclusions to older adults and other ethnic groups. Furthermore, although sensitivity analyses support the robustness of the study results, caution is still warranted when interpreting findings in specific subgroups. For example, in the normal waist circumference subgroup (n = 13,048, with a total of 337 events), when stratified by BFP quartiles, only 45 events occurred in the lowest quartile (Q1) group, and the HR for the Q4 group was as high as 3.81 compared to the Q1 group. This extremely high effect size may be partly attributable to random variation or the influence of outliers in the subgroup analysis; in particular, there is a potential risk of overfitting when adjusting for multiple metabolic covariates simultaneously. Therefore, although the data suggest that BFP can effectively identify high-risk individuals with “normal-weight obesity,” the specific HR values observed in this study (particularly for Q4 vs. Q1) should be considered hypothesis-generating in nature and require external validation in independent, larger-scale prospective cohorts. Finally, the lack of detailed data on dietary patterns and exercise intensity may, to some extent, limit a comprehensive analysis of lifestyle-mediated mechanisms. Future research should employ multiethnic cohorts, use DXA or CT to precisely quantify fat distribution, and conduct randomized controlled trials aimed at reducing BFP to verify causality and determine BFP intervention thresholds applicable to different populations.

In summary, this study established the independent and nonlinear predictive role of BFP in diabetes onset using large-scale cohort data. The identified 21.65% inflection point is not merely a statistical threshold but likely represents a critical turning point in the transition of adipose tissue from adaptive expansion to pathological remodeling. This finding provides a clear and actionable quantitative target for diabetes prevention: at the population level, preventing BFP from crossing the 21.65% threshold may be more cost-effective than simply pursuing weight loss. Future research should focus on developing simple BFP-based screening tools and, in combination with dietary interventions and resistance training, to verify whether reducing body fat percentage below this inflection point can effectively reverse insulin resistance and halt the progression of diabetes. This will not only help refine existing metabolic risk assessment systems but also provide important scientific evidence for achieving precision nutrition and personalized health management.

## Conclusion

In this large-scale retrospective cohort of Japanese adults, percent body fat (BFP) was confirmed as an independent risk factor for new-onset type 2 diabetes. Restricted cubic spline analysis revealed a significant nonlinear association, with an inflection point identified at 21.65%. Below this threshold, each 1% increase in BFP was associated with a markedly steeper increase in diabetes risk compared to levels above the threshold. This association remained robust in individuals with normal waist circumference and those without fatty liver disease, underscoring the superior sensitivity of BFP over BMI in detecting “normal-weight obesity.” Maintaining BFP below 21.65% may represent a practical and quantifiable target for diabetes prevention.

## Data Availability

The original contributions presented in the study are included in the article/supplementary material. Further inquiries can be directed to the corresponding author.
